# Perspectives on Artificial Intelligence–Generated Responses to Patient Messages

**DOI:** 10.1001/jamanetworkopen.2024.38535

**Published:** 2024-10-16

**Authors:** Jiyeong Kim, Michael L. Chen, Shawheen J. Rezaei, April S. Liang, Susan M. Seav, Sonia Onyeka, Julie J. Lee, Shivam C. Vedak, David Mui, Rayhan A. Lal, Michael A. Pfeffer, Christopher Sharp, Natalie M. Pageler, Steven M. Asch, Eleni Linos

**Affiliations:** 1 Center for Digital Health, School of Medicine, Stanford University, Stanford, California; 2 Division of Clinical Informatics, School of Medicine, Stanford University, Stanford, California; 3 Division of Endocrinology, Metabolism and Gerontology, Department of Medicine, School of Medicine, Stanford University, Stanford, California; 4 Primary Care and Population Health, Department of Medicine, School of Medicine, Stanford University, Stanford, California; 5 Division of Internal Medicine, Department of Medicine, School of Medicine, Stanford University, Stanford, California; 6 Stanford Diabetes Research Center, School of Medicine, Stanford University, Stanford, California; 7 Hospital Medicine, Department of Medicine, School of Medicine, Stanford University, Stanford, California; 8 Technology and Digital Solutions, School of Medicine, Stanford University, Stanford, California; 9 Stanford Health Care, Palo Alto, California; 10 Department of Pediatrics, School of Medicine, Stanford University, Stanford, California; 11 Information Services, Stanford Children’s Health, Palo Alto, California

## Abstract

This cross-sectional study of patient queries in US electronic health records examines laypersons’ satisfaction with answers generated with artificial intelligence (AI) compared with clinician responses, and whether results were concordant with clinician-determined quality of AI responses.

## Introduction

Generative artificial intelligence (AI) has the potential to assist clinicians in responding to patients’ messages.^1^ Although AI-generated responses were found to have acceptable quality with minimal risks of harm,^2,3,4^ the perspectives of laypeople toward AI responses have rarely been investigated despite their importance. Thus, we assessed laypersons’ satisfaction with the responses of AI vs clinicians-to-patient messages. Additionally, we examined if the clinician-determined quality of AI responses was concordant with satisfaction.

## Methods

In this cross-sectional study, out of 3 769 023 Patient Medical Advice Requests in electronic health records (EHRs), we screened 1089 clinical questions and included 59 messages (Figure). To mitigate possible selection bias, we developed and followed structured guidelines (eAppendix 1 in Supplement 1). Two generative AIs (ChatGPT-4, December 2023 version [OpenAI Inc] and Stanford Health Care and Stanford School of Medicine GPT, January 2024 version) created responses with and without prompt engineering (December 28, 2023, to January 31, 2024). Six licensed clinicians evaluated the AI and original clinician responses for information quality and empathy, using a 5-point Likert scale (with 1 indicating worst; 5, best). For satisfaction, 30 survey participants recruited through the Stanford Research Registry assessed the responses of AI (prompt-engineered Stanford GPT selected for highest quality AI responses) and clinicians (April 5 to June 10, 2024). Three individuals independently evaluated each response (with 1 being extremely dissatisfied; 5, extremely satisfied).^5^ To account for potential biases and variability of evaluators, we developed mixed models to compute effect estimates with standard errors for information quality, empathy, and satisfaction. We examined the association of response length with satisfaction using a multivariable linear regression accounting for age, sex, race, and ethnicity, where statistical significance was at *P* < .05. Analyses were conducted with SAS version 9.4 (SAS Institute Inc). We followed the Strengthening the Reporting of Observational Studies in Epidemiology (STROBE) reporting guideline. The institutional review board at Stanford University approved this study. We obtained systematically deidentified patient messages using Safe Harbor, and 2 researchers additionally verified no protected health information was present.

**Figure.**
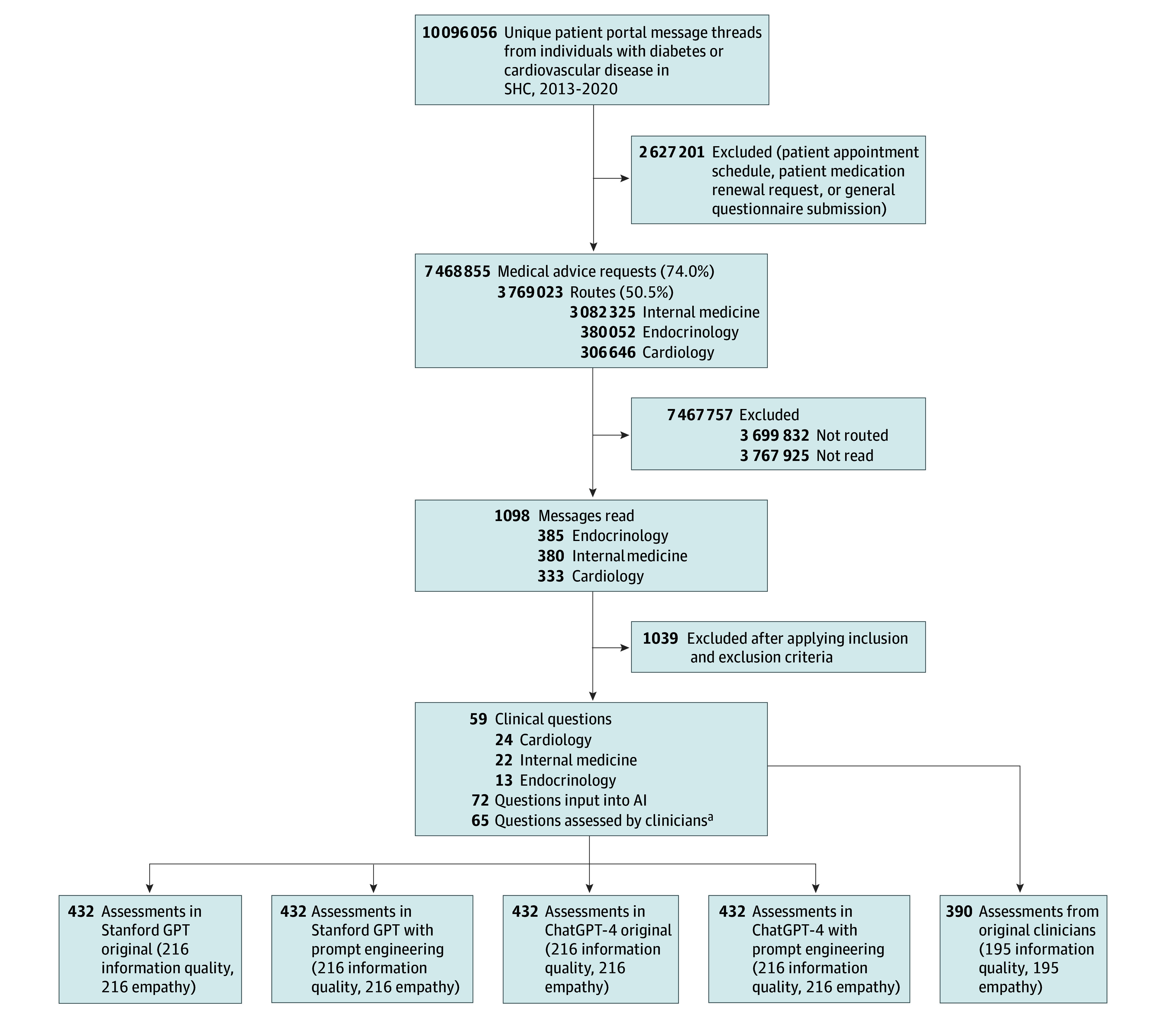
Study Design AI indicates artificial intelligence; LLM, large language model; SHC, Stanford Health Care. Messages were read in reverse chronological order. Each response was assessed by 3 clinicians for information quality and empathy (with LLM responses masked), and by 3 patient participants for satisfaction. ^a^When 1 message contained multiple separate topics, it was further divided into multiple questions: 11 original questions were divided into 24 questions for LLMs (ie, 59 questions became 72 questions), and 5 original questions were divided into 11 questions for clinicians’ responses (ie, from a total 59 questions to 65 questions).

## Results

A total of 2118 assessments for AI response quality and 408 assessments for satisfaction were included (Figure). Satisfaction estimates were higher with AI responses (3.96 [SE, 0.09]) than with clinicians’ responses (3.05 [SE, 0.09]), both overall (*P* < .001) and by specialty (Table). Satisfaction was highest with responses to cardiology questions from AI (4.09 [SE, 0.14]) while information quality and empathy were highest with responses to endocrinology questions. Clinicians’ responses were shorter (mean [SD] 254.37 [198.85] total characters) than AI responses (mean [SD] 1470.77 [391.83] total characters). Clinicians’ response length was associated with satisfaction overall (β = 0.23; *P* = .002) and in cardiovascular questions (β = 0.31; *P* = .02) while AI response length was not.

**Table.** Satisfaction of AI and Clinician Responses and Association With the Length of Responses

Division	AI^a^					Clinicians				
	Assessments, No.	Satisfaction estimate (SE)^b^	No. of characters, mean (SD)	Satisfaction and the length of response		Assessments, No.	Satisfaction estimate (SE)^b^	No. of characters, mean (SD)	Satisfaction and the length of response	
				Standardized β^c^	*P* value				Standardized β^c^	*P* value
Overall	213	3.96 (0.09)	1470.77 (391.83)	0.10	.16	195	3.05 (0.09)	254.37 (198.85)	0.23	.002
Cardiovascular	78	4.09 (0.14)	1559.04 (424.83)	0.068	.58	75	3.25 (0.14)	306.36 (221.09)	0.29	.02
Internal medicine	87	3.82 (0.13)	1314.72 (347.11)	0.037	.72	78	2.94 (0.14)	146.31 (109.43)	0.0056	.96
Endocrinology	48	4.00 (0.19)	1610.19 (330.87)	0.25	.08	42	2.90 (0.20)	362.21 (200.79)	0.31	.09

Abbreviations: AI, artificial intelligence.

^a^ Responses from Stanford GPT with prompts were assessed for satisfaction as it was graded as the best response in terms of information quality and empathy.

^b^ Satisfaction assessed on a 5-point scale, with 1 being the lowest and 5 the highest. *P*-values for the satisfactory estimate difference between AI vs clinicians were all *P* < .001 (overall, cardiovascular division, internal medicine division, and endocrinology division). The missing values were handled by missingness at random in the statistical model (mixed effect model).

^c^ To avoid too small β coefficients, we computed standardized β coefficients to present the strength of the effect of the length of response on the satisfaction estimate. The standardized β coefficient measures the changes in standard deviations (SD) of satisfaction estimates when 1 SD increases in the length of response. We adjusted it for age, sex, race, and ethnicity.

## Discussion

To our knowledge, this is the first study to assess satisfaction with AI-generated responses to patient-created medical questions in EHR. Satisfaction was consistently higher with AI-generated responses than with clinicians overall and by specialty. However, satisfaction was not necessarily concordant with the clinician-determined information quality and empathy. For example, satisfaction was highest with AI responses to cardiology questions while information quality and empathy were highest in endocrinology questions. Interestingly, clinicians’ response length was associated with satisfaction while AI’s response length was not. The findings suggest that the extreme brevity of responses could be a factor that lowers satisfaction in patient-clinician communication in EHR.

Study limitations include that satisfaction was assessed by survey participants rather than the patients who submitted the questions. Although original patients’ satisfaction might differ from that of survey participants, this study can provide the closest proxy of patients’ perspectives toward AI-generated responses. Future directions of the study include assessing satisfaction with AI responses in other settings (eg, regions and types of medical centers), study populations (eg, language and culture), and with larger samples across diverse specialties. Our study highlights the importance of incorporating patients as key stakeholders in the development and implementation of AI in patient-clinician communications to optimally integrate AI into practice.^6^

## Notes

Supplement 1. **eAppendix 1.** Inclusion and Exclusion Criteria to Sample Messages **eAppendix 2.** Prompt Engineering for AI Models

Supplement 2.
Data Sharing Statement
