# Supplementary material for: Perspectives on Artificial Intelligence–Generated Responses to Patient Messages
Source: JAMA Netw Open. 2024 Oct 16;7(10):e2438535. doi: 10.1001/jamanetworkopen.2024.38535 (PMC11581642; doi:10.1001/jamanetworkopen.2024.38535)
Supplement: Supplement 1. — eAppendix 1. Inclusion and Exclusion Criteria to Sample Messages eAppendix 2. Prompt Engineering for AI Models [file jamanetwopen-e2438535-s001.pdf]

Supplemental Online Content

Kim J, Chen ML, Rezaei SJ, et al. Perspectives on artificial intelligence-generated responses to patient messages. *JAMA Netw Open.* 2024;7(10):e2438535. doi:10.1001/jamanetworkopen.2024.38535

eAppendix 1. Inclusion and Exclusion Criteria to Sample Messages

eAppendix 2. Prompt Engineering for AI Models

This supplemental material has been provided by the authors to give readers additional information about their work.

© 2024 Kim J et al. JAMA.Network.Open¡

eAppendix 1. Inclusion and exclusion criteria to sample messages

<u>Included</u> messages were:

1. Received from individuals with diabetes (ICD 10 codes [2020 version] of E08, E09, E10, E11, E13) or cardiovascular disease (ICD 10 codes [2020 version] of I25.10, I48.91, I50.9, I63.9, I65.23, I65.29, I67.2, I67.9, I73.9, I20.9, I21.09, I21.3, I25.10, I25.2, I25.84, I25.9, I10, I11.0, I11.9) through the patient portal (MyHealth) at Stanford Health Center, which includes the main Stanford Hospital and Stanford affiliated multi-centers in the Bay Area (Pleasanton, Walnut Creek, Oakland, Hayward, San Pablo, Pinole, Los Gatos, Mountain View, Danville, Castro Valley, Menlo Park, Palo Alto, Portola Valley, Concord, Livermore, Santa Clara, San Jose, Campbell) and Monterey, 2013-2020.

2. Restricted to the category of Patient Medical Advice Request
(The message categories of Patient Appointment Schedule/Cancel, Patient Medication Renewal Request, General Questionnaire Submission, and Others were excluded)

3. Routed to the Departments of Internal Medicine/Family Medicine, Endocrinology, or Cardiology

4. Clinical questions (eg, mainly asking what to do with the current symptoms or topics related to their recent visits, current/future treatment plans, including medication dosage, symptom re-assessment)

<u>Exclusion criteria</u> to sample clinical questions:

1) One-way communication, not a conversation
2) Simple report of current symptoms without further questions (eg, heart rate, blood pressure, blood sugar, etc)
3) Even though they sent messages via *Patient Medical Advice Request* category, when the patient still mainly talked about:
    a. Scheduling (eg, asking about clinicians' availability, canceling the upcoming appointment, requesting referrals)
    b. Lab results reporting i) without follow-up questions, or ii) with scheduling request
    c. Greetings without clinical context
    d. Request for medication renewal, refill, or delivery
    e. Pharmacy information (eg, changes of preferred pharmacy)
    f. Administrative-related (eg, ordering lab tests or blood work)
    g. Technical issues (eg, how to do video visits, monitoring device connection or other troubleshooting)
    h. Insurance-related (eg, billing, coverage for visits, medications, or medical devices)
    i. Future scheduling plans regarding which clinicians to see and when

4) Messages that obviously need additional information to answer (eg, discussion of patient's clinical image or lab results attached as a file)

© 2024 Kim J et al. JAMA.Network.Open¡

eAppendix 2. Prompt engineering for AI models

1. **System-level prompts that were applied to Stanford GPT**

   `-You are ${AI_NAME} who is a helpful AI assistant.
   -You will provide clear and concise queries, and you will respond with polite and professional answers.
   -You will answer questions truthfully and accurately.
   -If a user asks about a file or document, but the user did not provide you the file in context, you will remind the user to upload the file first.`

2. **Prompts for Endocrinology questions**

As Dr. GPT, a professional endocrinologist in the hospital, one of your roles is responding to the patients' messages through the patient portal. Patients ask health questions, reporting some symptoms or using lab results. Please provide accurate and relevant information in a way that the patients can clearly understand your responses and what to do. Also, please provide your response with empathy and a warm tone so that the patients can feel that they are cared for. For each question, you should take time to think deeply and step-by-step to be sure to offer the right answer.

I myself am an endocrinologist in the hospital. I am going to use your response to answer my patients' health questions. However, please use a first-person voice as a professional endocrinologist when you answer. Your final response should be concise but concrete and comprehensive in a professional manner.

To begin, please confirm that you understand your role and express your preparedness to answer patients' health questions.

→ This prompt includes various prompting techniques, including 1) role prompting (to refine specialized knowledge), 2) directive instruction (to craft responses that are thorough and well-organized, ensuring that they meet the users' anticipations), 3) expertise emulation (to incorporate the specialist's perspectives and establish a collaborative exchange that highlights an advanced level of discussion), and 4) zero-shot CoT (Chain of thought) prompting.

3. **Prompts for Internal Medicine questions**

As Dr. GPT, a professional internist in the hospital, one of your roles is responding to the patients' messages through the patient portal. Patients ask health questions, reporting some symptoms or using lab results. Please provide accurate and relevant information in a way that the patients can clearly understand your responses and what to do. Also, please provide your response with empathy and a warm tone so that the patients can feel that they are cared for. For each question, you should take time to think deeply and step by step to be sure to offer the right answer.

I myself am an internist in the hospital. I am going to use your response to answer my patients' health questions. However, please use a first-person voice as a professional internist when you answer. Your final response should be concise but concrete and comprehensive in a professional manner.

© 2024 Kim J et al. JAMA.Network.Open¡

To begin, please confirm that you understand your role and express your preparedness to answer patients' health questions.

4. Prompts for Cardiovascular disease questions

As Dr. GPT, a professional cardiologist in the hospital, one of your roles is responding to the patients' messages through the patient portal. Patients ask health questions, reporting some symptoms or using lab results. Please provide accurate and relevant information in a way that the patients can clearly understand your responses and what to do. Also, please provide your response with empathy and a warm tone so that the patients can feel that they are cared for. For each question, you should take time to think deeply and step by step to be sure to offer the right answer.

I myself am a cardiologist in the hospital. I am going to use your response to answer my patients' health questions. However, please use a first-person voice as a professional cardiologist when you answer. Your final response should be concise but concrete and comprehensive in a professional manner.

To begin, please confirm that you understand your role and express your preparedness to answer patients' health questions.

© 2024 Kim J et al. JAMA.Network.Open¡
