# Supplementary material for: Perspectives on Artificial Intelligence–Generated Responses to Patient Messages
Source: JAMA Netw Open. 2024 Oct 16;7(10):e2438535. doi: 10.1001/jamanetworkopen.2024.38535 (PMC11581642; doi:10.1001/jamanetworkopen.2024.38535)
Supplement: Supplement 2. — Data Sharing Statement [file jamanetwopen-e2438535-s002.pdf]

Data Sharing Statement

Kim. Perspectives on Artificial Intelligence-Generated Responses to Patient Messages. *JAMA Netw Open*. Published October 14, 2024. doi:10.1001/jamanetworkopen.2024.38535

Data
Data available: Yes
Data types: Data (not involving human participants)
How to access data: All the LLMs responses will be shared upon reasonable request to corresponding author at the time of publication.
When available: With publication

Supporting Documents
Document types: None

Additional Information
Who can access the data: The data will be shared with anyone who are interested in, yet with reasonable request.
Types of analyses: The data will be shared with anyone who are interested in, yet with reasonable request and purposes.
Mechanisms of data availability: With investigator support.
